# Novel Point Mutations in the *NKX2.5* Gene in Pediatric Patients with Non-Familial Congenital Heart Disease

**DOI:** 10.3390/medicina54030046

**Published:** 2018-06-19

**Authors:** Mehri Khatami, Mansoureh Mazidi, Shabnam Taher, Mohammad Mehdi Heidari, Mehdi Hadadzadeh

**Affiliations:** 1 Department of Biology, Faculty of Science, Yazd University, Yazd 8915818411, Iran; m.mazidi69@yahoo.com (M.M.); shabnam_taher@stu.yazd.ac.ir (S.T.); heidarimm@yazd.ac.ir (M.M.H.); 2 Department of Cardiac Surgery, Afshar Hospital, Shahid Sadoughi University of Medical Sciences, Yazd 8915818411, Iran; mehad51@yahoo.com

**Keywords:** congenital heart disease, *NKX2.5*, mutation, PCR-SSCP

## Abstract

*Background and objective*: Congenital heart disease (CHD) is the most common birth abnormality in the structure or function of the heart that affects approximately 1% of all newborns. Despite its prevalence and clinical importance, the etiology of CHD remains mainly unknown. Somatic and germline mutations in cardiac specific transcription factor genes have been identified as the factors responsible for various forms of CHD, particularly ventricular septal defects (VSDs), tetralogy of Fallot (TOF), and atrial septal defects (ASDs). p. NKX2.5 is a homeodomain protein that controls many of the physiological processes in cardiac development including specification and proliferation of cardiac precursors. The aim of our study was to evaluate the *NKX2.5* gene mutations in sporadic pediatric patients with clinical diagnosis of congenital heart malformations. *Materials and methods*: In this study, we investigated mutations of the *NKX2.5* gene’s coding region in 105 Iranian pediatric patients with non-familial CHD by polymerase chain reaction-single stranded conformation polymorphism (PCR-SSCP) and direct sequencing. *Results*: We observed a total of four mutations, of which, two were novel DNA sequence variants in the coding region of exon 1 (c. 95 A > T and c. 93 A > T) and two others were previously reported as single-nucleotide polymorphisms (SNPs), namely rs72554028 (c. 2357 G > A) and rs3729753 (c. 606 G > C) in exon 2. Further, observed mutations are completely absent in normal healthy individuals (*n* = 92). *Conclusion*: These results suggest that *NKX2.5* mutations are highly rare in CHD patients. However, in silico analysis proves that c.95 A > T missense mutation in *NKX2.5* gene is probably pathogenic and may be contributing to the risk of sporadic CHD in the Iranian population.

## 1. Introduction

Congenital heart disease (CHD) is one of the most common types of human birth defects, which contributes substantially to non-infectious mortality. The incidence of moderate to severe forms of CHD is nearly 1% of all live births [1]. More than 30% of newborns who die of a birth defect have a cardiovascular malformation. CHD defines a large set of structural and functional defects that arise during cardiac embryogenesis [2]. Causes of CHD are often categorized into genetic and non-genetic factors that contribute to the very complex etiology of structural heart malformations, by disrupting highly regulated heart developmental processes. Non-genetic or environmental causes include infections, drugs, maternal exposures, and teratogens [3]. To find genetic factors in CHD, early studies of familial CHD were often shown different malformations such as ventricular septal defects (VSDs), tetralogy of Fallot (TOF) and atrial septal defects (ASDs) [4]. The haploinsufficiency in CHD causative genes suggests that gene dosage is critical for the normal heart formation. Well-recognized responsible genes in fetal heart development are regulated by those encoders for proteins that are a part of highly conserved transcriptional factors, including *NKX2.5*, *GATA4*, *JAG1*, *MEF2C*, and *TBX5* [5].

Among them, mutations in the *NKX2.5* homeobox gene (OMIM #600584, 108900) have been identified in many forms of familial and sporadic congenital heart disease and congenital hypothyroidism [6,7]. For the first time in 1998, Schott et al. reported gene mutations in homeobox transcription factor NKX2.5 [8]. Subsequently, the next investigators showed that somatic mutations in the *NKX2.5* gene are significant mechanisms of the pathogenesis of CHD [9,10]. Also, *NKX2.5* affects the synthesis of the other transcriptional complexes and is an important regulatory key in the differentiation and developmental processes in heart tissue. Loss of transcription factor p.NKX2.5 in mice leads to arrest, cardiac morphogenesis, and lethality due to impaired cardiac development [11]. Due to its critical role in transcriptional regulation and its interaction with other transcription factors in early cardiac development, *NKX2.5* has been a preferred candidate gene to identify the genetic determinants for congenital heart defects [12].

The human *NKX2.5* gene locates on chromosome 5q34, has 10,209 bp lengths and consists of two exons encoding a protein of 324 amino acids [13]. p. NKX2.5 is a highly conserved homeobox protein and contains one Tinman domain (TN) (residues 10–21), one homeodomain (residues 138–197), and one NK2 domain (residues 212–234) [14]. The most common CHD phenotypes associated with *NKX2.5* mutations are atrial septal defect (ASD), ventral septal defect (VSD), and tetralogy of Fallot (TOF). These different forms of congenital heart disease abnormalities are highly penetrant; however, some patients may have one form of the CHD alone. Commonly, patients suffer from many symptoms such as cardiac enlargement, arrhythmias, pulmonary hypertension, congestive heart failure, delayed fetal brain development, and even sudden cardiac death [15]. Nevertheless, the molecular etiology responsible for CHD in most patients remains largely unknown. To date, more than 40 mutations within the *NKX2.5* gene have been reported in patients with a variety of congenital heart malformations [14,16]. These findings strongly suggest that p.NKX2.5 plays an essential role in heart development and maturation, cardiac function, and morphogenesis [11].

In order to clarify whether the somatic *NKX2.5* mutations play an important role in Iranian pediatric patients with CHD, we screened *NKX2.5* gene mutations in peripheral blood from 105 sporadic patients with different CHD phenotypes and compared to 92 healthy control individuals using polymerase chain reaction-single stranded conformation polymorphism (PCR-SSCP) and DNA sequencing approaches. We found four heterozygote sequence alterations in the *NKX2.5* gene that were absent in a control population.

## 2. Materials and Methods

### 2.1. Patients

A total of 105 unrelated pediatric patients with congenital heart malformations and 92 healthy subjects for mutation analysis were included in our study. Patients were clinically evaluated by a pediatric cardiologist. All samples in this study were obtained from CHD patients undergoing cardiac surgery at the Afshar cardiac hospital (Yazd, Iran) and assessed by clinical history, physical examination, 12-lead electrocardiogram, and echocardiography. In addition, 92 unrelated healthy individuals from the same geographic area and without any family history of CHD were used as healthy controls (42 male and 50 female). The clinical records of all patients did not present any recognized genetic syndromes or even other congenital malformations. This study protocol was approved by the Ethics Committee of the Yazd University and all procedures in the present study were in accordance with the Helsinki Declaration of 1975, as revised in 2008 and ethical standards (IR.SSU.REC.1395.223 (August 2016)). After informed consent was obtained, a 5 mL peripheral blood sample was collected from each patient. Blood samples were acquired at the time of surgery. Genomic DNA was extracted from blood leukocytes using a DNA isolation kit (Qiagene Co., Tehran, Iran).

### 2.2. Mutation Screening

The two coding exons and adjacent intron sequences of the *NKX2.5* gene and their flanking introns were amplified using the polymerase chain reaction (PCR). The exon 1 was amplified using two pairs, but exon 2 was subdivided into three overlapping amplimers. In this way, exons were amplified by a total of eight PCR primers derived from the flanking introns (Table 1). Primers were designed by primer design software (Primer Premier 5.0; Premier Biosoft Inc., Palo Alto, CA, USA), based on human *NKX2.5* cDNA and genomic sequences reported in The National Center for Biotechnology Information (NCBI) and their secondary structure was examined with Gene Runner version 3.05 (Hastings Software Inc., Hastings, NY, USA, http://www.generunner.com). Polymerase chain reaction (PCR) was performed in a total volume of 25-μL containing 1× Polymerase chain reaction buffer, 50 ng genomic DNA, 2 pmol/each forward and reverse primers, 1.5 mM MgCl_2_, 0.5 mM each dNTPs, and 0.5 U Taq polymerase (Qiagen). The polymerase chain reaction program of exon 1 initiated with 4 min at 95 °C followed by 30 cycles of 35 s 95 °C, 35 s at 59 °C and 35 s at 72 °C, and finished with a 7 min extension time at 72 °C. For the exon 2, after an initial denaturation step at 95 °C during 4 min, 35 cycles were performed, each one consisting of 50 s at 95 °C, 35–45 s at 58.5 °C to 61.5 °C annealing temperatures (due to three different fragments in exon 2) and 40 s at 72 °C, followed by a final extension of 7 min at 72 °C. The amplified products were analyzed for size on a 1.5% agarose gel. For mutation screening, single-stranded conformation polymorphism (SSCP) analysis was performed in our patient and control samples. For single-stranded conformation polymorphism assay, 10 μL of polymerase chain reaction products were mixed with 7 μL single-stranded conformation polymorphism (SSCP) loading solution (80% formamide/10 mM NaOH/0.25% xylene cyanol FF/40% sucrose). Then DNA fragments were heat-denatured at 93 °C for 5 min and immediately chilled on ice for 3 min, and loaded onto a polyacrylamide gel (49:1 acrylamide to bis-acrylamide) (Sigma, Taufkirchen, Germany). The single-stranded conformation polymorphism was carried out with a Payapajoohesh vertical gel. The gel concentrations and running conditions were as follows: 6% polyacrylamide gel, 18 h, room temperature, 120 V. After the run, the gel was removed from the apparatus and the DNA bands were visualized with the silver staining method according to the standardized protocol. Samples showing an abnormal mobility profile within the single-stranded conformation polymorphism gel when compared with the healthy controls were submitted to direct sequencing.

### 2.3. Bioinformatics Analysis

The online multiple sequence alignment software ClustalW2 (http://www.ebi.ac.uk/tools/msa/clustalw2/) and sequence alignment was performed using the Standard Protein Blast (blastp) program available at the National Center for Biotechnology Information (NCBI) website (http://www.ncbi.nlm.nih.gov/Blastp). Also, polymorphism phenotyping v2 (PolyPhen-2) (http://genetics.bwh.harvard.edu/pph2/) and scale-invariant feature transform (SIFT) (http://sift.jcvi.org/) were used for prediction of the functional consequences of mutations and damaging effects of missense mutations on the protein sequence. Also, the PSI-blast based secondary structure PREDiction (PSIPRED) (http://bioinf.cs.ucl.ac.uk/psipred/) was used for secondary structure prediction in mutant protein. Finally, to determine the hydrophobicity or hydrophilicity scales in the proteins, a degree of protein hydrophobicity was assessed using a plot created by the Expert Protein Analysis System (ExPASy) Protscale tool (http://web.expasy.org/protocol).

### 2.4. Statistical Analysis

Using Fisher’s exact test (*X2*)*,* differences in the allele and genotype frequency of *NKX2.5* polymorphisms between our patient and control population were analyzed. In our results, a number of alleles tested was corrected and a *p* value of <0.05 was considered statistically significant.

## 3. Results

**Figure 1:**
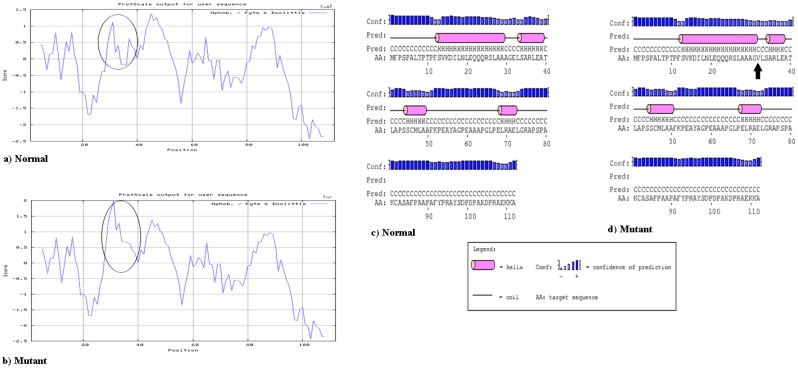
The Hydropathy Plot of the Amino Acids Sequence of normal (**a**) and mutant (**b**) NKX2.5 Protein. Secondary structure prediction in normal and mutant protein is indicated in (**c**,**d**) parts.

**Figure 2:**
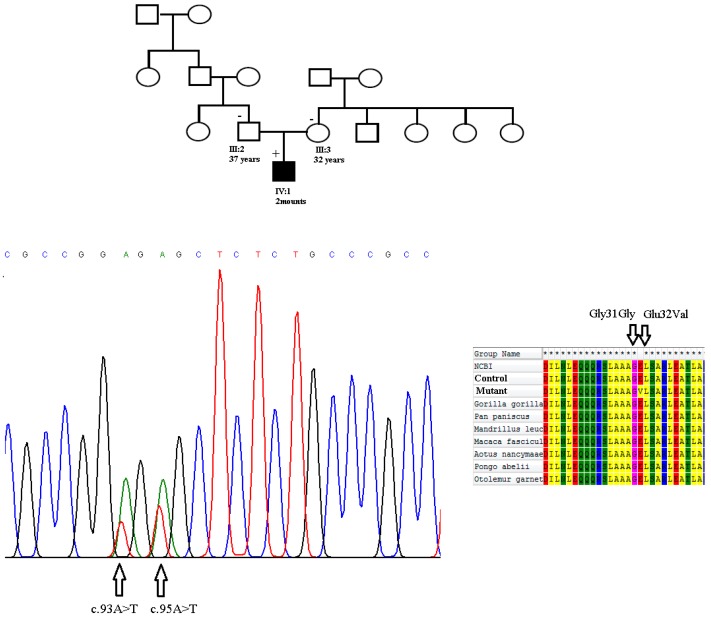
Sequence analysis of the genomic DNA of the patient revealed the heterozygous transition of an A to T at position 324 (p. Glu32Val) and a heterozygous synonym variation at 322 A > T in exon 1 *NKX2.5* gene in a child with Tetralogy of Fallot and ventricular septal defect (**left**). Multiple alignment of amino acids of human NKX2.5 protein with corresponding NKX2.5 sequences of different species. Positions of altered amino acids are indicated by black arrows (**right**).

In this study, a cohort of 105 unrelated pediatric patients with CHD was recruited and clinically evaluated against a cohort of 92 ethnically matched unrelated healthy subjects used as controls. In the patient group, 68 (64.76%) individuals had a ventral septal defect (VSD) and 25 (23.8%) suffered from atrial septal defect (ASD). Also, tetralogy of Fallot (TOF) was identified in 12 (11.42%) pediatric patients. Sixty seven were female (63.8%) and 38 were male (36.19%). The median age at the time of surgery was 2.8 years (range 0–4.5 years). The baseline clinical characteristics of the unrelated patients are shown in Table 2. A mutation analysis of the coding exons and exon/intron boundaries of *NKX2.5* gene was performed using polymerase chain reaction-single stranded conformation polymorphism (PCR-SSCP) and four heterozygous single nucleotide changes were identified in three unrelated patients: one novel non-synonymous variant and three synonymous variations were found in the NKX2.5 coding region (Table 3). The c.95 A > T missense mutation was heterozygous and for the first time was identified in a boy with Tetralogy of Fallot and a large ventricular septal defect. This sequence variant results in a Glutamic acid-to-Valine amino acid substitution in codon 32 of the protein (p.Glu32Val), and is located in exon 1, just outside of the Tinman domain (TN). This alteration in amino acid might impair the proper function of the NKX2.5 protein, because it changes the hydropathy determinants of the protein (hydropathy score was −3.500 for polar Glutamic acid which changes to 4.200 for nonpolar Valine) (Figure 1a,b). However, it was considered to be a significant missense mutation. Also, three separate synonymous sequence variations were found in our patients. The first is a novel single nucleotide polymorphism: Glycine 31 Glycine in exon 1, which was found in a two month old infant with Tetralogy of Fallot and a large ventricular septal defect. The patient was heterozygous for this nucleotide change. This patient was also heterozygous for the Glutamic acid 32 Valine mutation. The patient sequence chromatogram showing the detected heterozygous c.95 A > T and c.93 A > T variations in comparison to control sequence are shown in Figure 2. This child died during surgery. Both of the mutations have not been previously reported in various CHDs and were not found in any of the 92 healthy controls. Notably, these amino acids are highly conserved among several species. However, the parents of this patient were healthy and did not smoke and had no history of exposure to toxic chemicals. Family segregation analysis was performed for c.95 A > T and c.93 A > T novel variations in parents of this patient, but it revealed negative segregation with the disease in these cases (Figure 2). Also, for prediction of the protein stability and secondary structure, we performed several in-silico analyses. Our results showed that the Glutamic acid 32 Valine mutation is probably pathogenic. The prediction of secondary structures through Bioinformatics analysis showed that the residue Glutamic acid 32 is part of a coil region located in a conserved region among different mammalian species. The protein hydrophobicity plots present that the Glutamic acid 32 Valine mutation leads to partially changes in coil regions (Figure 1c,d).

The second synonymous variant was a previously described polymorphism (SNP), rs72554028 was a transition of a G to an A at nucleotide 2357 in exon 2 that predicts no changes at the protein level (p. Glu181Glu) and was identified as heterozygous in one patient (1.2 years old) with VSD. The third sequence variant, rs3729753 (c. G606C) in exon 2 was present in a girl patient (0.8 years old) with ASD and total anomalous pulmonary venous return. This polymorphism is a silent transversion of G to C at nucleotide 606 that does not induce changes of leucine in codon 202 of the protein (p. Leu202Leu). This patient was also heterozygous for this reported previously sequence variant. None of the sequence alterations identified in this study were present in the 92 normal controls. Based on a multiple sequence alignment of the corresponding amino acid sequence, all of these variants change highly conserved evolutionarily nucleotides among different species, suggesting that these sites are functionally important.

## 4. Discussion

**Figure 3:**
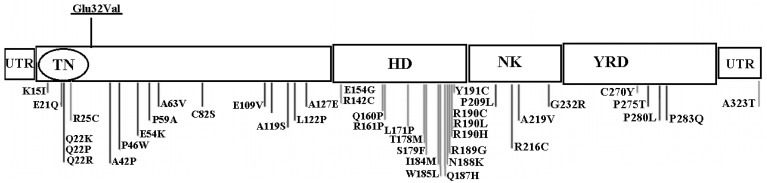
Previously published *NKX2.5* mutations in different functional domains of the NKX2.5 protein. Underlined Variant refers to novel missense variation reported in the current study. UTR = Untranslated region. TN = Tinman domain. HD = Homeodomain. NK = NK2 domain and YRD = Tyrosine rich domain.

Several studies have shown that *NKX2.5* gene plays critical roles in embryonic heart progenitor determination, cardiac morphogenesis, and conduction and contraction system development. *NKX2.5* gene does this by regulating the expression of several ion channel genes and controlling atrial septal formation [17,18]. Among vertebrates, *NKX2.5* is the most highly conserved gene and subject to transcriptional control via a complex series of cis-regulatory elements [19]. Because cardiac transcription factors are dosage-sensitive regulators during embryonic morphogenesis, haploinsufficiency in *NKX2.5*, due to a loss of functional mutations is associated with atrioventricular conduction defects and tetralogy of Fallot [20]. Huang et al. reported that reduced expression levels of the *NKX2.5* gene in a mutant protein could play significant roles in congenital heart defects [21]. To date, nearly 50 mutations of this gene have been recognized in various CHD phenotypes, including ventricular septal defects (VSDs), Tetralogy of Fallot (TOF), and atrial septal defects (ASDs) in different geographic areas (Figure 3) [11]. In Iran, very little research has been done on this gene. In one study, a High Resolution Melt (HRM) analysis and mutation scan of the *NKX2.5* gene in patients located in Kurdistan Province was conducted. It was found that there is only one polymorphism (A65G) in two atrial septal defect patients [22]. In another study, one synonymous variant (i.e., c. 543G > A) was identified in one patient from twenty-seven infants and children with tetralogy of Fallot [23]. According to our knowledge, the present study is the most comprehensive research on this gene in Iranian CHD patients. We analyzed the sequence of the *NKX2.5* coding region using DNA extracted from whole peripheral blood of 109 unrelated patients diagnosed with CHD in order to identify novel mutations. All subjects have no history of family marriages and have no migration history within three generations. We found four nucleotide changes, including two novel mutations in exon 1: c. 95 A > T heterozygous transversion (Glutamic acid 32Valine) and c. 93 A > T heterozygous synonymous variant (Glycine31Glycine), which both were found in a male child suffering from tetralogy of Fallot and a large ventricular septal defect who died during surgery and two known polymorphisms in exon 2: a silent mutation c. 2357G > A (rs72554028) and a synonymous variant c. 606G > C (rs3729753). Interestingly, in our study, the novel c. 95 A > T and c. 93 A > T changes were found in a two-month old boy with tetralogy of Fallot and large ventricular septal defect. Both mutations have never been previously reported in various CHDs or controls. In our study, these mutations were not identified in any of the ethnically matched subjects. c. 95 A > T mutation is located in the N-terminal of the homeodomain of p. NKX2.5 and results in the substitution of highly conserved polar glutamic acid at position 32 of the amino acid sequence with hydrophobic Valine. Where possible, familial segregation analysis was performed for c. 95 A > T and c. 93 A > T novel variations in parents of this patient, although the number of available family members has been often small and included the parents and the patient himself, but it revealed negative segregation with the disease in these cases. It is known that mutations in this prolific region of the *NKX2.5* gene are associated with the CHD. Tian et al. were identified by three mutations, G270A (Glu32Lys), G378A (Glu68Lys), and G390A (Glu72Lys) in the *NKX2.5* gene in a Chinese family with atrial septal defect (ASD) [24]. These mutations were not found in the 126 healthy controls, which suggests that these mutations play an important role in the pathogenesis of CHD. Also, Kasahara et al., Goldmuntz et al. and others demonstrated that protein expression in mutants of this region impaired DNA binding to dimeric sites and reduced minimal to absent transcriptional activation [25,26,27]. Also, results of the in-silico analysis, such as polymorphism phenotyping v2 (PolyPhen-2) and scale-invariant feature transform (SIFT) indicated that the c. 95 A > T genetic variant is probably damaging and associated with CHD. However, the functional effect of these mutations remains unresolved. The novel c. 93 A > T change is a silent mutation that has not yet been registered in the OMIM (Online Mendelian Inheritance in Man). Although this variation is a synonymous alteration, it is known that such mutations could play a role in the severity of the disease by transcriptional changes such as alternative splicing or exon skipping. Synonymous point mutations can disrupt an inappropriate splicing process by impairment in consensus exonic splicing enhancer or silencer regions as reported in a number of diseases including spinal muscular atrophy and breast cancer [28,29]. Further investigations in mRNA level must be done to determine the transcription defects in these patients.

The silent polymorphism c. 2357 G > A (rs72554028) has already been reported in a young woman with a cryptogenic stroke, Patent foramen ovale (PFO), and massive right-to-left shunt (RLS) by Belvis et al. and it has not been associated with the severity of the disease [30]. In our study, this polymorphism was only detected in one patient (1.2 years old) with ventricular septal defect and was not found in any other patients or healthy control subjects. Because there is no further literature for this polymorphism in CHD and our results also do not show a significant statistical association between this single nucleotide polymorphism with CHD, more data is needed to describe the effect of this polymorphism.

The relationships between sporadic atrial septal defects and ventricular septal defects in Chinese Yunnan population with c. 606G > C (rs3729753) SNPs of *NKX2.5* gene have been reported previously in 4.5% of controls by Cao et al. But their results do not provide evidence that the rs3729753 SNP in *NKX2.5* gene is associated with sporadic CHD in Chinese people [31]. Also, rs3729753 has already been reported in Chinese CHD patients by Zhang et al. and the allele frequency of this SNP has no significant differences between the 230 CHD patients and 200 controls [32]. In our study, it was present only in a girl patient (0.8 years old) with atrial septal defects and a total anomalous pulmonary venous return, albeit without a statistical significance.

Here, we analyzed exons 1 and 2 and the intron-exon boundaries of the *NKX2.5* gene and described two novel mutations and two known SNPs which occurs in Iranian pediatric patients with ventricular septal defects, tetralogy of Fallot, and atrial septal defects. None of these nucleotide variations were seen in healthy subjects. On the basis of the present results and previous findings, we propose that these genetic variations could be as susceptibility factors and responsible for congenital heart defects in Iranian patients.

### Limitations of the Study

In this study we investigated mutations of the *NKX2.5* gene’s coding region and the intron-exon boundaries in 105 Iranian pediatric patients with non-familial CHD by polymerase chain reaction-single stranded conformation polymorphism (PCR-SSCP) and direct sequencing. Limitations of the study should consider the need to assess the frequency of the mutations described in a larger cohort of individuals. Another limitation of our study group was the lack of information regarding family history of the CHD for all the study participants. In addition, functional studies are required to investigate the contribution of these novel variants to the CHD phenotype.

## 5. Conclusions

The novel c.95 A > T (Glu32Val) missense mutation in *NKX2.5* gene may be associated with non-familial CHDs with diverse clinical phenotypes in Iranian pediatric patients. However, the small sample size of this study does not allow us to prove such an association. Additional studies are required to identify the mechanisms by which this novel mutation leads to genomic instability and contributes to the risk of sporadic CHD in Iranian population. These findings expand the spectrum of mutations in the *NKX2.5* gene and provide new insight into the molecular mechanism involved in the pathogenesis of CHD.

## Figures and Tables

**Table 1:** Primers for the amplification of exons in *NKX2.5* gene.

Exon	Primer Sequences	Product Size (bp)
Exon 1	F: 5′-CCGCTTTCTGCCGCCCACC-3′	390
	R:5′-TCCTCCTCACCTTTCTTTTCG-3′	
Exon 2 (fragment 1)	F: 5′-CAAGCGTCTCTCTGCCTCTC-3′	242
	R: 5′-CTGCGTGGACGTGAGTTTC-3′	
Exon 2 (Fragment 2)	F: 5′-TGCTGAAACTCACGTCCAC-3′	230
	R: 5′-ATAACCGTAGGGATTGAGGC-3′	
Exon 2 (Fragment 3)	F: 5′-AACGCCTACCCCGCCTATC-3′	224
	R: 5′-CCAGGCTCGGATACCATGC-3′	

**Table 2:** Clinical characteristics of the 105 unrelated patients with CHD.

Clinical Characteristics	Number	Percentage or Range
Median Age (years)	2.8	0–4.5
Male:female	38:67	36.19:63.8
Distribution of different types of CHD		
Isolated ASD	25	23.8
Isolated VSD	68	64.76
Isolated TOF	12	11.42
ASD + VSD	6	5.71
ASD + TOF	1	0.95
VSD + TOF	2	1.90
ASD + VSD + TOF	1	0.95
Surgical treatment	65	61.9

CHD: Congenital Heart Defects, ASD: Atrial Septal Defect, VSD: Ventricular Septal Defect, TOF: Tetralogy of Fallot.

**Table 3:** Results of the mutation analysis in *NKX2.5* gene.

Genetic Findings	Region	Function	Amino-Acid Change	Novel/Reported
c.95A > T	Exon 1	Missense	Glu32Val	Novel
c.93A > T	Exon 1	Synonymous	Gly31Gly	Novel
c.2357G > A	Exon 2	Synonymous	Glu181Glu	Reported
rs72554028				
c.606G > C	Exon 2	Synonymous	Leu202Leu	Reported
rs3729753				
